# *N*′-(2-Methoxy­benzyl­idene)-2-nitro­benzo­hydrazide

**DOI:** 10.1107/S1600536809005753

**Published:** 2009-02-21

**Authors:** Ge-Jiang Xiao, Chao Wei

**Affiliations:** a School of Chemistry and Biological Engineering, Changsha University of Science and Technology, Changsha Hunan 410004, People’s Republic of China

## Abstract

The title compound, C_15_H_13_N_3_O_4_, was synthesized by the reaction of equimolar quanti­ties of 2-methoxy­benzaldehyde and 2-nitro­benzohydrazide in methanol. The dihedral angle between the two substituted benzene rings is 68.3 (2)°. In the crystal structure, inversion dimers linked by pairs of N—H⋯O hydrogen bonds occur.

## Related literature

For the pharmacological properties of hydrazone compounds, see: Beraldo & Gambino (2004 ▶). For related structures, see: Galić *et al.* (2001 ▶); Richardson & Bernhardt (1999 ▶); Ali *et al.* (2004 ▶). For bond length data, see: Allen *et al.* (1987 ▶).
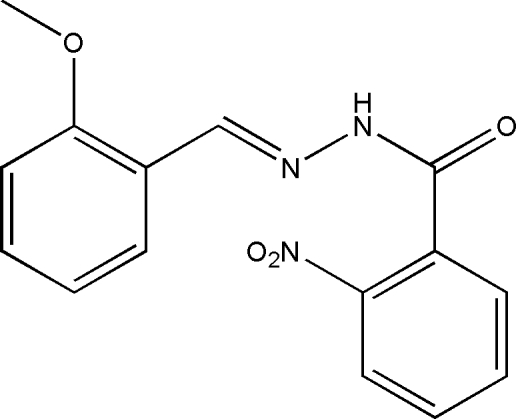


## Experimental

### 

#### Crystal data

C_15_H_13_N_3_O_4_
*M* *_r_* = 299.28
Triclinic, 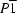

*a* = 7.491 (2) Å
*b* = 9.427 (3) Å
*c* = 10.977 (3) Å
α = 91.748 (4)°
β = 106.218 (4)°
γ = 92.221 (4)°
*V* = 743.1 (4) Å^3^
*Z* = 2
Mo *K*α radiation
μ = 0.10 mm^−1^
*T* = 298 K
0.23 × 0.23 × 0.22 mm

#### Data collection

Bruker SMART 1000 CCD area-detector diffractometer
Absorption correction: multi-scan (*SADABS*; Bruker, 2001 ▶) *T*_min_ = 0.978, *T*_max_ = 0.979
6232 measured reflections
3140 independent reflections
2018 reflections with *I* > 2σ(*I*)
*R*_int_ = 0.022

#### Refinement

*R*[*F* ^2^ > 2σ(*F* ^2^)] = 0.048
*wR*(*F* ^2^) = 0.132
*S* = 1.03
3140 reflections
203 parameters
1 restraint
H atoms treated by a mixture of independent and constrained refinement
Δρ_max_ = 0.14 e Å^−3^
Δρ_min_ = −0.20 e Å^−3^

### 

Data collection: *SMART* (Bruker, 2007 ▶); cell refinement: *SAINT* (Bruker, 2007 ▶); data reduction: *SAINT*; program(s) used to solve structure: *SHELXTL* (Sheldrick, 2008 ▶); program(s) used to refine structure: *SHELXTL*; molecular graphics: *SHELXTL*; software used to prepare material for publication: *SHELXTL*.

## Supplementary Material

Crystal structure: contains datablocks global, I. DOI: 10.1107/S1600536809005753/sj2574sup1.cif

Structure factors: contains datablocks I. DOI: 10.1107/S1600536809005753/sj2574Isup2.hkl

Additional supplementary materials:  crystallographic information; 3D view; checkCIF report

## Figures and Tables

**Table 1:** Hydrogen-bond geometry (Å, °)

*D*—H⋯*A*	*D*—H	H⋯*A*	*D*⋯*A*	*D*—H⋯*A*
N1—H1⋯O1^i^	0.910 (9)	1.943 (10)	2.844 (2)	170.3 (18)

Symmetry code: (i) 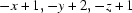.

**Fig. 1.**
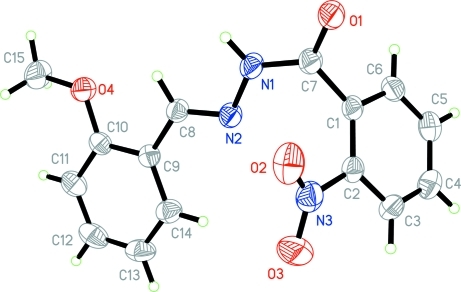
The molecular structure of (I), showing 30% probability displacement ellipsoids and the atom-numbering scheme.

**Fig. 2.**
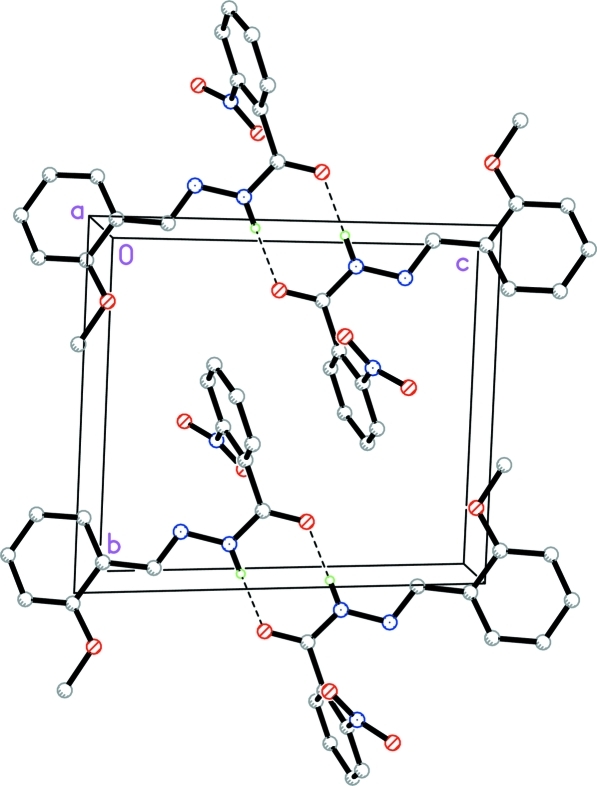
The crystal packing of (I), viewed along the a axis. Dashed lines indicate hydrogen bonds.

## supplementary crystallographic information

### Comment

Hydrazone compounds have received considerable attention due to their pharmacological properties (Beraldo & Gambino, 2004). In the last few years, the crystal structures and properties of a series of hydrazone compounds have been reported (Galić *et al.*, 2001; Richardson & Bernhardt, 1999; Ali *et al.*, 2004). As a continuation of work on these compounds, we report here the structure of the title compound, (I) Fig. 1.

In (I), the dihedral angle between the C1—C6 and C9—C14 benzene rings is 111.7 (2)° while that between the O2—N3—O3 nitro plane and the plane of the C1—C6 benzene ring is 26.7 (2)°. Bond lengths in the compound are found to have normal values (Allen *et al.*, 1987). The methoxy group is coplanar with the C9—C14 benzene ring, with a C15—O4—C10—C11 torsion angle of -3.2 (2)°.

In the crystal packing, adjacent molecules are linked through intermolecular N1–H1···O1 hydrogen bonds (Table 1), forming dimers (Fig. 2).

### Experimental

The title compound was synthesized by the reaction of equimolar quantities (1.0 mmol each) of 2-methoxybenzaldehyde and 2-nitrobenzohydrazide in methanol (100 ml) for 3 h at room temperature. The solution was kept in air for a few days, forming colorless block-like crystals of the compound.

### Refinement

The N-bound H atom was located in a difference Fourier map and was refined with an N–H distance restraint of 0.90 (1) Å. C-bound H atoms were placed in calculated positions (C–H = 0.93–0.96 Å) and refined using a riding model with *U*_iso_(H) = 1.2*U*_eq_(C) and 1.5*U*_eq_(C15). Crystals were small and weakly diffracting which explains the relatively low data fraction.

### Crystal data

**Table:**

C_15_H_13_N_3_O_4_	*Z* = 2
*M**_r_* = 299.28	*F*(000) = 312
Triclinic, *P*1	*D*_x_ = 1.338 Mg m^−^^3^
Hall symbol: -P 1	Mo *K*α radiation, λ = 0.71073 Å
*a* = 7.491 (2) Å	Cell parameters from 1428 reflections
*b* = 9.427 (3) Å	θ = 2.8–24.9°
*c* = 10.977 (3) Å	µ = 0.10 mm^−^^1^
α = 91.748 (4)°	*T* = 298 K
β = 106.218 (4)°	Block, colorless
γ = 92.221 (4)°	0.23 × 0.23 × 0.22 mm
*V* = 743.1 (4) Å^3^	

### Data collection

**Table:**

Bruker SMART 1000 CCD area-detector diffractometer	3140 independent reflections
Radiation source: fine-focus sealed tube	2018 reflections with *I* > 2σ(*I*)
graphite	*R*_int_ = 0.022
ω scans	θ_max_ = 27.0°, θ_min_ = 1.9°
Absorption correction: multi-scan (*SADABS*; Bruker, 2001)	*h* = −9→9
*T*_min_ = 0.978, *T*_max_ = 0.979	*k* = −12→11
6232 measured reflections	*l* = −13→14

### Refinement

**Table:**

Refinement on *F*^2^	Primary atom site location: structure-invariant direct methods
Least-squares matrix: full	Secondary atom site location: difference Fourier map
*R*[*F*^2^ > 2σ(*F*^2^)] = 0.048	Hydrogen site location: inferred from neighbouring sites
*wR*(*F*^2^) = 0.132	H atoms treated by a mixture of independent and constrained refinement
*S* = 1.03	*w* = 1/[σ^2^(*F*_o_^2^) + (0.0615*P*)^2^ + 0.0142*P*] where *P* = (*F*_o_^2^ + 2*F*_c_^2^)/3
3140 reflections	(Δ/σ)_max_ = 0.001
203 parameters	Δρ_max_ = 0.14 e Å^−^^3^
1 restraint	Δρ_min_ = −0.20 e Å^−^^3^

### Special details

**Table:**

Geometry. All e.s.d.'s (except the e.s.d. in the dihedral angle between two l.s. planes) are estimated using the full covariance matrix. The cell e.s.d.'s are taken into account individually in the estimation of e.s.d.'s in distances, angles and torsion angles; correlations between e.s.d.'s in cell parameters are only used when they are defined by crystal symmetry. An approximate (isotropic) treatment of cell e.s.d.'s is used for estimating e.s.d.'s involving l.s. planes.
Refinement. Refinement of *F*^2^ against ALL reflections. The weighted *R*-factor *wR* and goodness of fit *S* are based on *F*^2^, conventional *R*-factors *R* are based on *F*, with *F* set to zero for negative *F*^2^. The threshold expression of *F*^2^ > σ(*F*^2^) is used only for calculating *R*-factors(gt) *etc*. and is not relevant to the choice of reflections for refinement. *R*-factors based on *F*^2^ are statistically about twice as large as those based on *F*, and *R*- factors based on ALL data will be even larger.

### Fractional atomic coordinates and isotropic or equivalent isotropic displacement parameters (Å^2^)

**Table:**

	*x*	*y*	*z*	*U*_iso_*/*U*_eq_	
O1	0.6095 (2)	0.83513 (13)	0.54829 (12)	0.0763 (4)	
O2	0.1921 (2)	0.69215 (17)	0.37644 (16)	0.0874 (5)	
O3	0.1059 (2)	0.54863 (19)	0.2124 (2)	0.1155 (7)	
O4	0.32257 (18)	1.20069 (12)	0.01171 (11)	0.0635 (4)	
N1	0.4749 (2)	0.90208 (15)	0.35161 (13)	0.0609 (4)	
N2	0.3966 (2)	0.86740 (14)	0.22397 (12)	0.0526 (4)	
N3	0.2239 (2)	0.60480 (19)	0.30207 (19)	0.0714 (5)	
C1	0.5624 (2)	0.65719 (17)	0.38490 (14)	0.0479 (4)	
C2	0.4158 (2)	0.56387 (18)	0.32220 (16)	0.0504 (4)	
C3	0.4423 (3)	0.43069 (18)	0.27844 (17)	0.0620 (5)	
H3	0.3408	0.3709	0.2360	0.074*	
C4	0.6193 (3)	0.3869 (2)	0.29785 (19)	0.0686 (5)	
H4	0.6392	0.2967	0.2690	0.082*	
C5	0.7670 (3)	0.4760 (2)	0.35974 (18)	0.0663 (5)	
H5	0.8875	0.4463	0.3725	0.080*	
C6	0.7390 (2)	0.6095 (2)	0.40340 (16)	0.0590 (5)	
H6	0.8411	0.6684	0.4461	0.071*	
C7	0.5456 (3)	0.80422 (18)	0.43443 (16)	0.0559 (5)	
C8	0.3456 (2)	0.97399 (17)	0.15700 (15)	0.0496 (4)	
H8	0.3691	1.0645	0.1958	0.060*	
C9	0.2516 (2)	0.95924 (17)	0.02188 (15)	0.0473 (4)	
C10	0.2377 (2)	1.07927 (19)	−0.05146 (15)	0.0507 (4)	
C11	0.1419 (3)	1.0699 (2)	−0.17899 (17)	0.0675 (5)	
H11	0.1328	1.1499	−0.2275	0.081*	
C12	0.0606 (3)	0.9428 (3)	−0.2335 (2)	0.0777 (6)	
H12	−0.0041	0.9371	−0.3193	0.093*	
C13	0.0727 (3)	0.8231 (2)	−0.1637 (2)	0.0735 (6)	
H13	0.0170	0.7370	−0.2020	0.088*	
C14	0.1680 (3)	0.8317 (2)	−0.03657 (18)	0.0618 (5)	
H14	0.1763	0.7508	0.0107	0.074*	
C15	0.3219 (4)	1.3252 (2)	−0.0573 (2)	0.0835 (7)	
H15A	0.3822	1.3086	−0.1227	0.125*	
H15B	0.3874	1.4019	−0.0009	0.125*	
H15C	0.1958	1.3497	−0.0953	0.125*	
H1	0.462 (3)	0.9897 (13)	0.3841 (18)	0.080*	

### Atomic displacement parameters (Å^2^)

**Table:**

	*U*^11^	*U*^22^	*U*^33^	*U*^12^	*U*^13^	*U*^23^
O1	0.1187 (12)	0.0584 (8)	0.0431 (8)	0.0124 (8)	0.0071 (7)	0.0026 (6)
O2	0.0841 (11)	0.0920 (11)	0.1047 (12)	0.0271 (9)	0.0521 (9)	0.0251 (10)
O3	0.0594 (10)	0.1026 (13)	0.1606 (18)	−0.0046 (9)	−0.0059 (11)	−0.0071 (12)
O4	0.0816 (9)	0.0535 (7)	0.0498 (7)	−0.0058 (6)	0.0095 (6)	0.0122 (6)
N1	0.0937 (12)	0.0441 (8)	0.0412 (8)	0.0091 (8)	0.0119 (8)	0.0035 (6)
N2	0.0670 (9)	0.0485 (8)	0.0426 (8)	0.0070 (7)	0.0149 (7)	0.0036 (6)
N3	0.0590 (11)	0.0633 (11)	0.0941 (14)	0.0018 (9)	0.0236 (10)	0.0200 (10)
C1	0.0598 (11)	0.0455 (9)	0.0382 (9)	0.0046 (8)	0.0122 (7)	0.0101 (7)
C2	0.0515 (10)	0.0488 (10)	0.0536 (10)	0.0057 (8)	0.0180 (8)	0.0126 (8)
C3	0.0713 (13)	0.0459 (10)	0.0673 (12)	−0.0034 (9)	0.0175 (10)	0.0045 (9)
C4	0.0838 (15)	0.0526 (11)	0.0747 (13)	0.0144 (10)	0.0292 (11)	0.0060 (10)
C5	0.0629 (12)	0.0700 (13)	0.0697 (13)	0.0201 (10)	0.0213 (10)	0.0160 (10)
C6	0.0552 (11)	0.0629 (12)	0.0541 (11)	0.0023 (9)	0.0067 (8)	0.0105 (9)
C7	0.0734 (12)	0.0496 (10)	0.0427 (10)	0.0033 (9)	0.0127 (8)	0.0069 (8)
C8	0.0599 (10)	0.0437 (9)	0.0454 (10)	0.0036 (8)	0.0148 (8)	0.0032 (8)
C9	0.0486 (9)	0.0490 (10)	0.0459 (9)	0.0047 (7)	0.0153 (7)	0.0012 (8)
C10	0.0483 (10)	0.0598 (11)	0.0436 (9)	0.0040 (8)	0.0118 (7)	0.0036 (8)
C11	0.0670 (12)	0.0854 (15)	0.0466 (11)	0.0043 (11)	0.0096 (9)	0.0082 (10)
C12	0.0665 (13)	0.1108 (18)	0.0486 (11)	0.0036 (12)	0.0059 (9)	−0.0106 (12)
C13	0.0657 (13)	0.0799 (15)	0.0706 (14)	−0.0082 (11)	0.0166 (10)	−0.0269 (12)
C14	0.0641 (12)	0.0577 (11)	0.0635 (12)	0.0007 (9)	0.0191 (9)	−0.0070 (9)
C15	0.1165 (18)	0.0631 (13)	0.0682 (14)	−0.0017 (12)	0.0200 (12)	0.0229 (11)

### Geometric parameters (Å, °)

**Table:**

O1—C7	1.2283 (19)	C5—C6	1.377 (3)
O2—N3	1.218 (2)	C5—H5	0.9300
O3—N3	1.215 (2)	C6—H6	0.9300
O4—C10	1.358 (2)	C8—C9	1.453 (2)
O4—C15	1.416 (2)	C8—H8	0.9300
N1—C7	1.333 (2)	C9—C14	1.385 (2)
N1—N2	1.3828 (19)	C9—C10	1.399 (2)
N1—H1	0.910 (9)	C10—C11	1.382 (2)
N2—C8	1.270 (2)	C11—C12	1.364 (3)
N3—C2	1.461 (2)	C11—H11	0.9300
C1—C6	1.376 (2)	C12—C13	1.375 (3)
C1—C2	1.386 (2)	C12—H12	0.9300
C1—C7	1.496 (2)	C13—C14	1.377 (3)
C2—C3	1.372 (2)	C13—H13	0.9300
C3—C4	1.365 (3)	C14—H14	0.9300
C3—H3	0.9300	C15—H15A	0.9600
C4—C5	1.366 (3)	C15—H15B	0.9600
C4—H4	0.9300	C15—H15C	0.9600
			
C10—O4—C15	118.70 (14)	N1—C7—C1	118.62 (15)
C7—N1—N2	121.79 (14)	N2—C8—C9	122.23 (15)
C7—N1—H1	117.1 (13)	N2—C8—H8	118.9
N2—N1—H1	120.3 (13)	C9—C8—H8	118.9
C8—N2—N1	113.81 (14)	C14—C9—C10	118.43 (16)
O3—N3—O2	124.2 (2)	C14—C9—C8	122.47 (16)
O3—N3—C2	117.72 (19)	C10—C9—C8	119.04 (15)
O2—N3—C2	118.09 (18)	O4—C10—C11	124.23 (17)
C6—C1—C2	116.74 (16)	O4—C10—C9	115.40 (14)
C6—C1—C7	117.41 (16)	C11—C10—C9	120.37 (17)
C2—C1—C7	125.85 (16)	C12—C11—C10	119.7 (2)
C3—C2—C1	122.53 (17)	C12—C11—H11	120.2
C3—C2—N3	117.31 (17)	C10—C11—H11	120.2
C1—C2—N3	120.16 (16)	C11—C12—C13	121.14 (19)
C4—C3—C2	119.23 (18)	C11—C12—H12	119.4
C4—C3—H3	120.4	C13—C12—H12	119.4
C2—C3—H3	120.4	C12—C13—C14	119.44 (19)
C3—C4—C5	119.76 (18)	C12—C13—H13	120.3
C3—C4—H4	120.1	C14—C13—H13	120.3
C5—C4—H4	120.1	C13—C14—C9	120.9 (2)
C4—C5—C6	120.57 (19)	C13—C14—H14	119.5
C4—C5—H5	119.7	C9—C14—H14	119.5
C6—C5—H5	119.7	O4—C15—H15A	109.5
C1—C6—C5	121.16 (18)	O4—C15—H15B	109.5
C1—C6—H6	119.4	H15A—C15—H15B	109.5
C5—C6—H6	119.4	O4—C15—H15C	109.5
O1—C7—N1	121.37 (16)	H15A—C15—H15C	109.5
O1—C7—C1	119.77 (15)	H15B—C15—H15C	109.5

### Hydrogen-bond geometry (Å, °)

**Table:**

*D*—H···*A*	*D*—H	H···*A*	*D*···*A*	*D*—H···*A*
N1—H1···O1^i^	0.91 (1)	1.94 (1)	2.844 (2)	170 (2)

Symmetry codes: (i) −*x*+1, −*y*+2, −*z*+1.
